# The Flipside of Eradicating a Disease; Human African Trypanosomiasis in a Woman in Rural Democratic Republic of Congo: A Case Report

**DOI:** 10.3390/tropicalmed4040142

**Published:** 2019-12-11

**Authors:** Junior Mudji, Jonathan Benhamou, Erick Mwamba-Miaka, Christian Burri, Johannes Blum

**Affiliations:** 1 Hôpital Evangélique de Vanga, Vanga Mission, B.P. 4728 Kinshasa 2, Congo; mudjijunior@gmail.com; 2 Unit of Clinical Pharmacology and Pharmacovigilance, Protestant University of Congo, B.P. 4745 Kinshasa 2, Congo; 3 Swiss Tropical and Public Health Institute, 4002 Basel, Switzerland; jonathan.benhamou@hotmail.com (J.B.); christian.burri@swisstph.ch (C.B.); 4 University of Basel, 4001 Basel, Switzerland; 5 Programme National de Lutte contre la Trypanosomiase Humaine Africaine (PNLTHA), Kinshasa 2, Congo; erickmwamb2002@yahoo.fr

**Keywords:** Human African Trypanosomiasis (HAT), mydriasis, neurological signs, eradication, re-emergence

## Abstract

Human African Trypanosomiasis (HAT) is a neglected disease caused by the protozoan parasites Trypanosoma brucei and transmitted by tsetse flies that progresses in two phases. Symptoms in the first phase include fever, headaches, pruritus, lymphadenopathy, and in certain cases, hepato- and splenomegaly. Neurological disorders such as sleep disorder, aggressive behavior, logorrhea, psychotic reactions, and mood changes are signs of the second stage of the disease. Diagnosis follows complex algorithms, including serological testing and microscopy. Our case report illustrates the course of events of a 41-year old woman with sleep disorder, among other neurological symptoms, whose diagnosis was made seven months after the onset of symptoms. The patient had consulted two different hospitals in Kinshasa and was on the verge of being discharged from a third due to negative laboratory test results. This case report highlights the challenges that may arise when a disease is on the verge of eradication.

## 1. Background

Human African Trypanosomiasis (HAT) is a neglected disease that progresses in two phases. Symptoms in the first phase include fever, headaches, pruritus, lymphadenopathy, and, in certain cases, hepato- and spleno- megaly. Neurological disorders such as sleep disorder, aggressive behavior, logorrhea, psychotic reactions, and mood changes are signs of the second stage of the disease [1,2]. Laboratory tests are needed for a definitive confirmation of the diagnosis. Our case report illustrates the case of a 41-year old woman with sleep disorder, among other neurological symptoms, whose diagnosis was made seven months after onset of symptoms. The patient had consulted two different hospitals in Kinshasa and was on the verge of being discharged from the third due to negative laboratory test results and an alarming lack of knowledge concerning this very treatable disease. This case report attempts to highlight the problems that may arise when a disease is on the verge of eradication.

## 2. Clinical presentation

A 41-year old woman working as a teacher presented to the emergency department of Vanga Hospital with sleep and behavioral disorders, along with tremors in both upper limbs. Her next of kin reported that she had been sick for seven months. Symptoms started with headaches, and one month later, sleep disorder, aggressive behavior, and mood changes appeared. In addition, her husband reported unquantified weight loss, sporadic fever episodes, logorrhea, and urinary incontinence in the past two months. The patient had no known medical history of mental illness. She is married and lives with her husband and two children. Tobacco, alcohol, or illicit drug use were denied. Until 2007, the patient had lived in a village in western Democratic Republic of Congo (DRC), where HAT is endemic. Since then, the family has moved to a part of Kinshasa, which, incidentally, is also endemic for HAT. Before consulting Vanga Hospital, the patient had been to two different hospitals in Kinshasa, both of which had treated her for malaria without any improvement in symptoms. 

**Figure 1:**
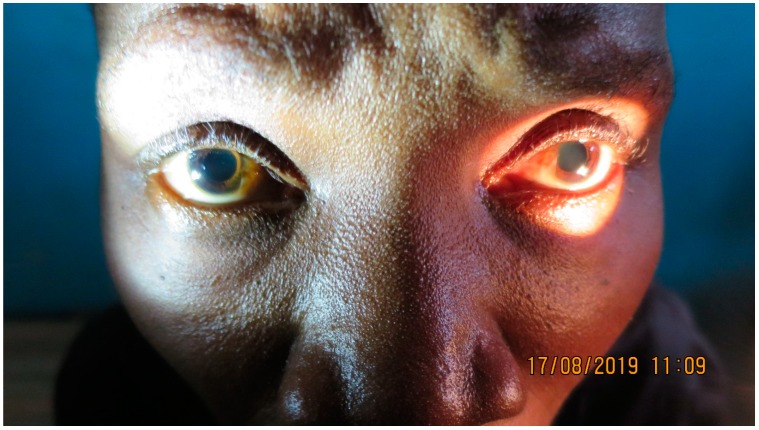
Areflective bilateral mydriasis in HAT patient.

Upon arrival, her vital parameters were as follows: pulse rate was 76 beats per minute (bpm), blood pressure was 80/60 mmHg, respiratory rate was 16 breaths per minute, and corporeal temperature was 36.7 °C. The patient’s consciousness level teetered between fast asleep and wide awake. When awake, she was fully oriented and had no loss of memory. The results of the physical examination were as follows: respiratory excursions were full and symmetrical, lungs resonant to percussion, and a normal, vesicular breath sound in all fields; no rales, rhonchi, wheezes, or rubs were present. A cardiovascular examination showed a regular rate and rhythm with no extra sounds or murmurs; jugular venous pressure was normal. Concerning the abdomen, bowel sounds were normal. Superficial and deep palpation did not reveal any organomegaly or masses, and no evidence of direct or rebound tenderness was found. Neurological examination showed clear and fluent speech. No nuchal rigidity was found, and Kernig’s and Brudzinski’s signs were negative. Cranial nerve examination showed no abnormalities aside from an areflexic bilateral mydriasis (see Figure 1). While cranial nerve palsies and ocular nerve symptoms have been described in the past [3], an isolated areflective bilateral mydriasis has, to our knowledge, never been reported in HAT patients. Fundoscopy was normal without signs of elevated intracranial pressure or inflammation. Slight symmetrical muscle weakness (4/5) was seen in both the upper and lower limbs with flexors, extensors, abductors, and adductors all being affected; muscle tone, however, was normal. Sensory function was intact in the fingers and toes. Her reflexes were 1+ and symmetric at the biceps, triceps, and ankles, and 3+ and symmetric at the patella; Babinski’s sign was negative. The patient showed a slow and uneven gait, without any signs of asymmetry, dragging of toes on the ground, or loss of balance. Trendelenburg sign was negative. An examination of her coordination showed normal diadochokinesia, a negative Romberg’s sign, and a slightly slow and uncoordinated Finger-to-Nose-Test without any signs of tremor.

The first laboratory tests showed negative thick drop and blood smear for malaria and trypanosomiasis, a thrice negative Card Agglutination Test for Trypanosomiasis (CATT), and a positive Rapid Plasma Reagin (RPR) for syphilis. The attending physician confirmed the diagnosis of neurosyphilis and a treatment with ceftriaxone was planned. The differential diagnosis of syphilis and HAT is challenging, because both diseases have a broad clinical spectrum of neurological and psychiatric findings, and false positive results of RPR have been reported. Being that the patient had spent several days in hospital without receiving proper treatment, the family wanted to remove her from the hospital’s care. Had the staff doctor not intervened at that moment, asking for a lumbar puncture, the patient would have been sent home without a certain diagnosis, and therefore, without receiving adequate treatment. Examination of the spinal fluid showed 105 white blood cells per mm^3^ and the presence of live trypanosomes, confirming the diagnosis of stage-2 HAT.

**Figure 2:**
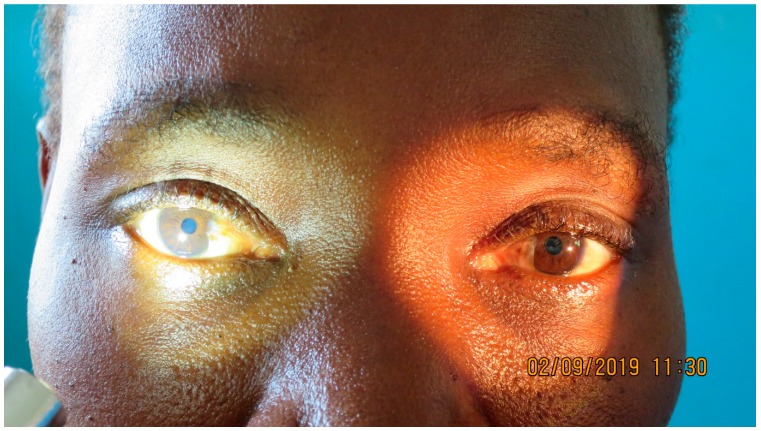
The same HAT patients at the end of treatment with normal pupillary reaction.

Treatment with albendazol and nifurtimox-eflornithine combination therapy (NECT) was immediately started. Following a full 10-day cycle of treatment, the patient was re-evaluated. She reported a drastic improvement in her general state of health. The behavioral disorders, somnolence, and tremor in both upper limbs had disappeared. Upon physical examination, the patient showed clear signs of improvement. The areflexic bilateral mydriasis had disappeared under treatment (see Figure 2), as did the muscle weakness in all four limbs. Her reflexes were 2+ and symmetric at the biceps, triceps, and ankles, and remained at 3+ at the patella. Gait and coordination exams, including a Finger-to-Nose-Test, showed no signs of impairment.

## 3. Discussion

**Figure 3:**
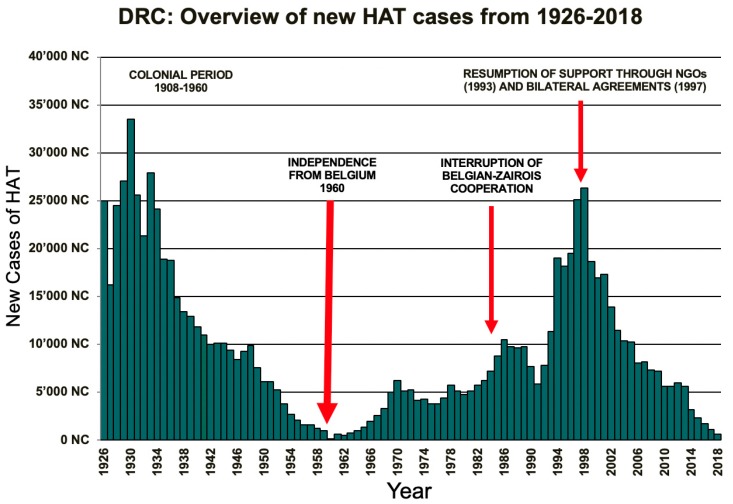
Overview of new HAT cases in the DRC from 1926-2018. Figure from the Programme National de Lutte contre la Trypanosomiase Humaine Africaine (PNLTHA), Kinshasa, DR of Congo, 2018.

What is alarming about this case, is the number of misdiagnoses within the three hospital stays the patient had. Thanks to numerous efforts during colonial times, HAT was nearly eradicated in the DRC (see Figure 3). Following the DRC’s independence, political unrest and the interruption of the Belgian-Zairois cooperation led to a drastic increase in new cases. Thanks to national and international efforts following the re-emergence of HAT as a major health concern in the DRC, the prevalence of new cases decreased from 26,318 in 1998 to 660 in 2018. This success brings with it one main downside, namely, that with decreasing prevalence of HAT, younger doctors and nurses do not see many cases, and therefore, lack appropriate awareness and knowledge about the disease. The thrice false negative CATT is worrying. There are two possible explanations: First, CATT is only available in kits with 50 aliquots of reagents, which remain stable for 3–5 days, and it is possible that an expired reagent was used. Second, with the decreasing number of patients, laboratory personnel do not frequently see HAT cases, and are therefore, more error prone. In the three months following the diagnosis and treatment of the aforementioned patient, four more cases were diagnosed in Vanga Hospital, all in the second stage of the illness. All four patients consulted numerous hospitals before coming to Vanga. More worryingly, one of the four patients was kept in a psychiatric clinic and put on an antipsychotic treatment for two months.

## 4. Conclusions

HAT was almost eradicated in the DRC in the 1960s, but a civil war and a lack of attention to and funding for the disease led to a resurgence. To prevent this from happening again, young hospital personnel, especially in a country like the DRC, where HAT is endemic, require proper education, repetition training, and adequate funding. Continued public information is paramount to maintaining awareness of this disease. In this respect, the National Day of Commemoration of Human African Trypanosomiasis in the DRC on 30 January 2020, with the participation of the Head of State and the Minister of Health, is an important and commendable example.

We received written informed consent from the patient to use the pictures above as well as write about and publish her case.
